# Facility‐Level Factors Associated With Aspiration Pneumonia in Japanese Geriatric Health Service Settings: A Nationwide Cross‐Sectional Study

**DOI:** 10.1111/ggi.70410

**Published:** 2026-02-19

**Authors:** Xinze Wu, Tatsuma Okazaki, Jiro Okochi, Satoru Ebihara

**Affiliations:** ^1^ Department of Rehabilitation Medicine Tohoku University Graduate School of Medicine Sendai Japan; ^2^ Geriatric Health Services Faculty Tatsumanosato Daito Japan

**Keywords:** aspiration pneumonia, long‐term care facilities, occupational therapy

## Abstract

**Background:**

Aspiration pneumonia (AP) is a leading cause of morbidity and mortality in older adults. Facility‐level determinants of AP in long‐term care settings remain poorly understood. In Japan's long‐term care insurance system, geriatric health service facilities differ in their staff composition, care processes, and swallowing support systems, which may influence AP incidence.

**Methods:**

We conducted a nationwide, facility‐level cross‐sectional study using data from the 2024 Survey on Eating and Swallowing Support conducted by the Japan Association of Geriatric Health Service Facilities. Of the 454 respondents, 445 facilities were included. The facilities were categorized as super‐enhanced, enhanced, add‐on, or basic. Facility characteristics, staffing composition, nutritional and swallowing management practices, and reimbursement‐based care add‐ons were compared across facility types. Multivariate logistic regression was used to identify the risk factors associated with AP occurrence.

**Results:**

Facility type, staff composition, and care resources varied significantly. The multivariable model revealed that a history of AP (OR = 45.138, 95% CI 16.937–120.292; *p* < 0.001) and aspiration events (OR = 9.280, 95% CI 4.215–20.116; *p* < 0.001) were strongly associated with AP. Facilities lacking occupational therapists had a higher risk of AP (OR = 4.875, 95% CI 1.708–13.909; *p* = 0.003).

**Conclusions:**

Facilities characterized by frequent aspiration events or a history of AP tended to have a higher incidence of AP. The absence of occupational therapists was associated with an increased facility‐level risk of AP, suggesting that organizational differences in care management may influence AP in long‐term care settings.

## Graphical abstract

We conducted a nationwide, facility‐level cross‐sectional study. The incidence of aspiration pneumonia (AP) was compared among four facility types (super‐enhanced, enhanced, add‐on, and basic). Frequent aspiration, a history of AP, and lack of occupational therapists were associated with AP occurrence in long‐term care facilities.

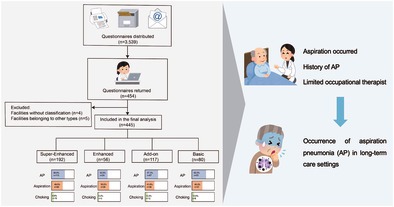

## 1 Introduction

Pneumonia continues to be a major health issue worldwide, affecting at‐risk individuals across older age categories [1]. Aspiration pneumonia (AP) is a distinct form of pneumonia that develops when oropharyngeal or upper gastrointestinal secretions containing microorganisms are aspirated into the lower respiratory tract, including the larynx and trachea [2]. The number of patients with pneumonia is increasing with an increase in the population of older adults, and AP accounts for the majority of cases, especially in individuals aged ≥ 65 years [3]. Therefore, AP was added as a category of cause of death to the 2017 Ministry of Health, Labor and Welfare report [3]. The number of deaths due to AP was 35 740, which accounted for a mortality rate of 28.7% and ranked seventh among the leading causes of death [3]. Although the disease does not lead to death and is cured, a decline in physical function and an increase in the amount of assistance required for daily activities are inevitable [3, 4, 5]. However, the concept itself remains vague worldwide, and global awareness is lacking; thus, neither diagnostic nor treatment strategies have been clearly established [6, 7]. Therefore, understanding the pathology of AP, early diagnosis, and establishment of an appropriate treatment system are urgent clinical issues.

Despite growing clinical attention to AP risk screening and bedside swallowing management, far less is known about the organizational determinants of AP‐related outcomes in long‐term care [8]. Japan's long‐term care insurance (LTCI) system reports that geriatric health service facilities are classified into distinct reimbursement‐based facility types (super‐enhanced, enhanced, add‐on, and basic, etc.) [9] that differ in staffing mix, care processes, and formalization of multidisciplinary swallowing support. These structural features plausibly influence the implementation of meal rounds, oral care, texture‐modified diets, and the rehabilitation provided by speech–language pathologists, physical therapists, and occupational therapists. Such elements may modify the facility‐level burdens of aspirations and AP. However, previous studies have largely focused on resident‐level risk factors, with limited facility‐level evidence comparing AP‐related outcomes across facility types after adjusting for case mix and staffing. To address this gap, we conducted a nationwide facility‐level analysis to test whether LTCI facility type was associated with the occurrence of AP at the facility level, independent of care‐need severity, bed capacity, staffing composition, and nutrition or swallowing support practices.

However, evidence directly comparing the preventive effectiveness of different types of facilities remains limited. Therefore, clarifying how institutional characteristics relate to AP occurrence is essential for establishing optimal care models and guiding health policies to reduce pneumonia‐related morbidity and mortality in an aging population. We hypothesized that facilities with enhanced organizational functions would exhibit lower odds of developing AP after adjustment.

## 2 Materials and Methods

### 2.1 Study Design and Data Source

This study was a retrospective, cross‐sectional observational study based on the facility‐level dataset of the “Survey on Eating and Swallowing Support” conducted in FY2024 by the Japan Association of Geriatric Health Service Facilities (JAGHSF). The original survey was a nationwide census targeting all 3539 geriatric health service facilities in Japan. Questionnaires were mailed to each facility, and responses were collected by mail, fax, or e‐mail. For the facility survey, return of the questionnaire was regarded as implied consent (*n* = 454). Only the facility‐level dataset was used; resident‐level questionnaires were not analyzed. From the returned facility questionnaires, we first excluded four records without a facility‐type classification and subsequently excluded five facilities whose types were outside the four predefined categories because of their small numbers, yielding a final analytic sample of 445 facilities (Figure S1). This study was approved by the Ethics Committee of Tohoku University (approval no. 2025‐01‐369).

### 2.2 Facility Classification

Facilities were classified according to the reimbursement category claimed as of November 1, 2024. This classification is an administrative variable officially defined within the LTCI system in Japan, which reflects differences in facility functions, staffing requirements, and service provision related to home‐return support. Under the LTCI system, geriatric health service facilities are reimbursed based on their structural capacity to support residents' rehabilitation and transition back to community living. The classification used in the present study represents a hierarchical framework, with higher categories indicating greater emphasis on rehabilitation services, multidisciplinary staffing, and home‐return support. In the facility questionnaire, respondents selected one category from predefined options. The classification is as follows:

- Super‐enhanced type (Cho‐kyoka‐gata): A subset of enhanced facilities that additionally claim the Home‐Return/Home‐Care Support Function Add‐on (Level II).
- Enhanced type (Kyoka‐gata): Facilities claiming an enhanced basic service fee for home‐return support.
- Add‐on type (Kasan‐gata): A subset of basic facilities that claim the Home‐Return/Home‐Care Support Function Add‐on (Level I).
- Basic type (Kihon‐gata): Facilities claiming the basic service fee.
- Other type (Sonota‐gata): Facilities claiming the “other” basic service fee.
- Long‐term care medical type (Ryouyou‐gata): Long‐term care medical geriatric health service facilities.

Each facility was assigned a single category according to its reimbursement status. This classification system has been widely adopted in national surveys to capture structural differences in facility functions and staffing. As described above, we analyzed four facility types only: super‐enhanced, enhanced, add‐on, and basic.

### 2.3 Definition of AP

The definition of AP in this study followed the 2024 Japanese Respiratory Society (JRS) Guidelines for Adult Pneumonia, which define AP as “pneumonia occurring in hosts at risk of aspiration due to impaired swallowing or gastroesophageal dysfunction.”

Since no universally accepted diagnostic standard is available, a comprehensive and pragmatic definition was applied in this study, integrating the criteria used in previous Japanese clinical and epidemiological studies. In practice, AP included cases with a witnessed or strongly suspected aspiration episode; pneumonia occurring in patients with clinical swallowing dysfunction or pharyngeal disorder; and pneumonia developing in individuals with established dysphagia, bed confinement, oral dysfunction, or gastroesophageal reflux without other identifiable causes. In addition, cases presenting with radiographic gravity‐dependent infiltrates together with inflammatory signs (fever, leukocytosis, purulent sputum, or elevated C‐reactive protein levels) and documented aspiration risk factors were also regarded as AP [10, 11, 12, 13]. Facility‐reported AP cases encompassed both clinically suspected and radiologically confirmed events consistent with the above criteria.

The present nationwide facility survey revealed that each participating institution recorded cases of AP based on their internal clinical judgment, consistent with these definitions, including both physician‐diagnosed and nursing‐reported cases occurring within the previous 3 months.

### 2.4 Covariates

Data were self‐reported by participating facilities through the nationwide survey. Only the facility‐level dataset was used for the present analysis, and resident‐level data were not included. The facility questionnaire collected information on structural and process‐related characteristics.

Structural characteristics included the total number of residents, the distribution of care‐need levels, the weighted mean care‐need level (calculated by multiplying each care level by its corresponding resident count, summing the products, and dividing by the total number of residents). The care‐level indicator used in this study is defined within the LTCI system and reflects the level of care needs and service intensity required by residents. This classification is routinely used in administrative and epidemiological studies as a proxy for institutional care demand and care complexity. Staff composition by profession was also collected, including physicians, dentists, nursing staff, certified care workers, care staff, registered dietitians, nutritionists, dental hygienists, speech–language pathologists, physical therapists, and occupational therapists. For each profession, facilities were categorized into quartiles based on the distribution of the number of staff in that profession across all facilities, such that the lowest quartile represented facilities with the fewest staff and the highest quartile represented those with the most staff for that profession.

Process‐related variables included the occurrence of aspiration and choking incidents within the preceding 3 months, the number of staple food texture categories provided, and the number of residents receiving enteral nutrition. In addition, facilities reported whether they had any history of AP occurring more than 3 months prior to the survey, which did not include AP events within the preceding 3‐month outcome window.

Moreover, reimbursement‐based indicators of nutritional and swallowing management under the Japanese LTCI system were included as facility‐level process variables. These comprised the nutritional management enhancement add‐on, oral intake transition add‐on, oral intake maintenance add‐ons (I and II), oral hygiene management add‐ons (I and II), and the nutritional collaboration add‐on at readmission. These add‐ons reflect the presence and intensity of structured, multidisciplinary nutritional and swallowing management systems at the facility level.

### 2.5 Statistical Analysis

Continuous variables are presented as mean ± standard deviation (SD) or median (interquartile range), as appropriate. Comparisons across facility types were performed using one‐way analysis of variance (ANOVA) for normally distributed variables and the Kruskal–Walli's test for non‐normally distributed variables. Categorical variables are presented as counts and percentages and were compared using the *χ* ^2^ test. Supplementary descriptive analyses stratified by AP status were conducted to compare facility‐level characteristics between facilities with and without reported AP and to provide additional context for the main regression analyses; detailed facility classifications and reimbursement‐based add‐on information are also presented in the Supplementary Tables.

Multivariable logistic regression analyses were performed to identify facility‐level factors. A hierarchical modeling strategy was adopted. Facility type was first entered as the primary exposure variable. The number of residents and the weighted mean care‐need level were included in all models as a priori adjustment variables to account for facility size and resident case‐mix. Variables showing statistically significant differences in the univariable analyses were entered into the multivariable model. The other covariates included staff composition (nurses, certified care workers, registered dietitians, dental hygienists, speech–language pathologists, physical therapists, and occupational therapists), nutritional support, aspiration, choking incidents, history of AP, and range of staple food texture options. Reimbursement‐based nutritional and swallowing management add‐ons were examined in supplementary models and are reported separately in Table S5. Backward stepwise selection was then applied to derive the final model, with variables sequentially removed if they did not meet the retention criterion (*p* > 0.05). Odds ratios (ORs) with 95% confidence intervals (CIs) were calculated for variables retained in the final model. Multicollinearity was assessed using variance inflation factors (VIF), with values of < 5 considered acceptable. *p* < 0.05 was considered statistically significant. Statistical analyses were primarily performed using IBM SPSS Statistics (version 29.0; IBM Corp., Armonk, NY, USA). To account for potential small‐sample bias and separation issues, Firth's bias‐reduced logistic regression analysis was conducted using R (version 4.3.3; R Foundation for Statistical Computing, Vienna, Austria).

## 3 Results

### 3.1 Baseline Characteristics of Facilities

The number of residents did not differ significantly across the facility types (*p* = 0.554). In contrast, resident characteristics varied, with significantly fewer individuals at care need levels 4 and 5 in the add‐on and basic facilities than in the super‐enhanced facilities (*p* < 0.001 and *p* = 0.003, respectively). The mean care level was highest in super‐enhanced facilities, suggesting that they tend to admit residents with greater care dependency (*p* < 0.001) (Table 1).

**TABLE 1:** Basic characteristics across facility types.

	Super enhanced	Enhanced	Add‐on	Basic	*p*
Care level 1 (*n*)	8.70 ± 5.42	10.00 ± 6.76	10.53 ± 5.90**	11.80 ± 7.44**	0.001
Care level 2 (*n*)	15.53 ± 6.23	15.52 ± 7.08	18.32 ± 21.12	17.20 ± 7.99	0.227
Care level 3 (*n*)	13.80 ± 6.83	12.82 ± 6.44	11.38 ± 6.74	10.84 ± 8.16	0.330
Care level 4 (*n*)	25.32 ± 9.55	24.66 ± 10.78	22.09 ± 10.83**	19.81 ± 10.48**	< 0.001
Care level 5 (*n*)	13.80 ± 6.83	12.82 ± 6.44	11.38 ± 6.74**	10.84 ± 8.16**	0.003
Number of residents (*n*)	84.37 ± 22.56	83.84 ± 27.08	81.80 ± 35.80	79.26 ± 27.73	0.554
Mean care level	3.23 ± 0.29	3.16 ± 0.35	3.05 ± 0.33*	2.99 ± 0.38**	< 0.001

*Note:* Data are presented as mean ± SD.

* *p* < 0.05.

** *p* < 0.01 vs. super enhanced type.

### 3.2 Staff Composition Across Facility Types

Marked differences were observed in the composition of healthcare professionals across facilities. The number of nursing staff was significantly higher in the super‐enhanced type, whereas add‐on and basic facilities tended to employ fewer nurses (*p* = 0.009). Similarly, the proportion of certified care workers decreased in less specialized facilities (*p* < 0.001). The availability of registered dietitians, dental hygienists, speech–language pathologists, physical therapists, and occupational therapists varied substantially, with super‐enhanced and enhanced facilities demonstrating greater staffing resources (*p* < 0.001) (Table 2).

**TABLE 2:** Comparison of staffing categories across facility types.

	Super enhanced	Enhanced	Add‐on	Basic	*p*
Physicians *n* (%)					0.768
< 1 person	15 (7.9)	8 (14.3)	10 (8.7)	9 (11.5)	
1 to < 2 persons	130 (83.8)	44 (78.6)	98 (85.2)	62 (79.5)	
≥ 2 persons	16 (8.4)	4 (7.1)	7 (6.1)	7 (9.0)	
Dentists *n* (%)					0.459
< 1 person	169 (98.3)	53 (98.1)	107 (100.0)	73 (100.0)	
1 to < 2 persons	1 (0.6)	1 (1.9)	0 (0.0)	0 (0.0)	
≥ 2 persons	2 (1.2)	0 (0.0)	0 (0.0)	0 (0.0)	
Nursing staff *n* (%)					0.009
< 6 persons	21 (10.9)	11 (20.0)	24 (21.2)	14 (17.7)	
6 to < 9 persons	46 (24.0)	10 (18.2)	39 (34.5)	30 (38.0)	
9 to < 12 persons	78 (40.6)	21 (38.2)	34 (30.1)	20 (25.3)	
≥ 12 persons	47 (24.5)	13 (23.6)	16 (14.2)	15 (19.0)	
Certified care workers *n* (%)					< 0.001
< 10 persons	9 (4.7)	4 (7.3)	15 (13.3)	12 (15.2)	
10 to < 20 persons	38 (20.0)	15 (27.3)	42 (37.2)	34 (43.0)	
20 to < 30 persons	93 (48.9)	26 (47.3)	41 (36.3)	22 (27.8)	
30 to < 40 persons	38 (20.0)	6 (10.9)	13 (11.5)	8 (10.1)	
≥ 40 persons	12 (6.3)	4 (7.3)	2 (1.8)	3 (3.8)	
Care staff *n* (%)					0.224
< 10 persons	138 (74.6)	44 (80.0)	90 (82.6)	54 (68.4)	
10 to < 20 persons	41 (22.2)	10 (18.2)	18 (16.5)	20 (25.3)	
≥ 20 persons	6 (3.2)	1 (1.8)	1 (0.9)	5 (6.3)	
Registered dietitians *n* (%)					< 0.001
0 persons	1 (0.5)	0 (0.0)	6 (5.2)	2 (2.5)	
0 to < 1 person	1 (0.5)	1 (1.8)	2 (1.7)	0 (0.0)	
1 to < 2 persons	87 (45.5)	24 (42.9)	60 (51.7)	58 (72.5)	
≥ 2 persons	102 (53.4)	31 (55.4)	48 (41.4)	20 (25.0)	
Nutritionists *n* (%)					0.263
0 persons	148 (86.0)	45 (83.3)	85 (80.2)	58 (80.6)	
0 to < 1 person	17 (9.9)	5 (9.3)	19 (17.9)	8 (11.1)	
1 to < 2 persons	1 (0.6)	0 (0.0)	0 (0.0)	0 (0.0)	
≥ 2 persons	6 (3.5)	4 (7.4)	2 (1.9)	6 (8.3)	
Dental hygienists *n* (%)					< 0.001
0 persons	142 (79.8)	48 (90.6)	102 (94.4)	65 (90.3)	
0 to < 1 person	2 (1.1)	2 (3.8)	0 (0.0)	0 (0.0)	
1 to < 2 persons	25 (14.0)	3 (5.7)	6 (5.6)	7 (9.7)	
≥ 2 persons	9 (5.1)	0 (0.0)	0 (0.0)	0 (0.0)	
Speech‐language pathologists *n* (%)					< 0.001
0 persons	60 (33.0)	26 (47.3)	67 (62.0)	60 (82.2)	
0 to < 1 person	9 (4.9)	3 (5.5)	1 (0.9)	1 (1.4)	
1 to < 2 persons	83 (45.6)	21 (38.2)	35 (32.4)	9 (12.3)	
≥ 2 persons	30 (16.5)	5 (9.1)	5 (4.6)	3 (4.1)	
Physical therapists *n* (%)					< 0.001
0 persons	5 (2.6)	0 (0.0)	11 (9.8)	5 (6.5)	
0 to < 1 person	2 (1.1)	0 (0.0)	1 (0.9)	1 (1.3)	
1 to < 2 persons	14 (7.4)	6 (10.7)	28 (25.0)	19 (24.7)	
≥ 2 persons	168 (88.9)	50 (89.3)	72 (64.3)	52 (67.5)	
Occupational therapists *n* (%)					< 0.001
0 persons	14 (7.4)	5 (8.9)	17 (15.5)	21 (27.3)	
0 to < 1 person	3 (1.6)	3 (5.4)	1 (0.9)	3 (3.9)	
1 to < 2 persons	33 (17.63)	12 (21.4)	30 (27.3)	28 (36.4)	
≥ 2 persons	138 (73.4)	36 (64.3)	62 (56.4)	25 (32.5)	

### 3.3 Incidence of AP‐Related Events Across Facility Types

The crude incidences of AP and aspiration demonstrated no significant differences across facility types. Approximately half of the facilities reported a history of AP, with no significant differences observed among facility types (Table 3).

**TABLE 3:** Comparison of AP‐related events across facility types.

	Super enhanced	Enhanced	Add‐on	Basic	*p*
AP *n* (%)	113 (58.9)	24 (42.9)	67 (57.3)	50 (62.5)	0.120
History of AP *n* (%)	99 (51.6)	22 (39.3)	51 (43.6)	41 (51.2)	0.269
Aspiration *n* (%)	95 (49.5)	28 (50.0)	48 (41.0)	37 (46.3)	0.501
Choking *n* (%)	19 (9.9)	3 (5.4)	12 (10.3)	8 (10.0)	0.739

Abbreviation: AP, aspiration pneumonia.

Supplementary analyses stratified by AP status revealed that the number of residents, mean care‐need level, facility type, and staff composition were comparable between AP and non‐AP facilities (Table S1). Similar results were obtained when the facilities were stratified according to aspiration status (Table S2). In the analyses according to choking incidents, the overall staffing distributions were also comparable, except for certified care workers (*p* = 0.023). Other professional categories did not differ significantly (Table S3).

### 3.4 Nutritional Support, Modified Diets and Implementation Across Facility Types

Enteral feeding was most common in the super‐enhanced facilities, with significantly fewer cases observed in the add‐on and basic facilities (*p* = 0.004). No significant differences were observed for parenteral nutrition (*p* = 0.510) (Table 4). Regarding diet provision, the number of modified staple food types was significantly lower in basic facilities than in super‐enhanced facilities (*p* = 0.004), whereas differences in dish provision and snack availability were not statistically significant (Table 4).

**TABLE 4:** Comparison of nutritional management and food provision across facility types.

	Super enhanced	Enhanced	Add‐on	Basic	*p*
Nutritional management					
Enteral nutrition	4.40 ± 3.97	3.73 ± 3.26	2.85 ± 3.07**	3.23 ± 4.55*	0.004
Parenteral nutrition	0.27 ± 1.25	0.23 ± 1.51	0.08 ± 0.36	0.17 ± 0.71	0.510
Food provision					
Number of staple food types	3.76 ± 0.52	3.77 ± 0.47	3.70 ± 0.48	3.50 ± 0.75**	0.004
Number of main and side dish types	7.67 ± 3.02	6.70 ± 2.96*	8.04 ± 3.07^#^	7.56 ± 3.45	0.067
Snacks provided by the facility	1.53 ± 0.65	1.77 ± 0.74*	1.64 ± 0.70	1.65 ± 0.69	0.109
Snacks provided by family members	1.29 ± 0.54	1.36 ± 0.52	1.23 ± 0.42	1.19 ± 0.42	0.177

*Note:* Data are presented as mean ± SD.

Abbreviation: AP, aspiration pneumonia.

* *p* < 0.05.

** *p* < 0.01 compared with super enhanced type.

^#^ *p* < 0.05, compared with enhanced type.

Across facility types, the distribution of nutrition‐ and oral‐management add‐ons differed for several items (Table S4). Nutritional management enhancement showed a significant difference across facility types (*p* < 0.001). Oral intake transition did not differ significantly among facility types (*p* = 0.153). In contrast, oral intake maintenance I and oral intake maintenance II both differed significantly across facility types (*p* < 0.001). Oral hygiene management I did not differ significantly (*p* = 0.369), whereas oral hygiene management II showed a significant difference across facility types (*p* < 0.001). The nutritional collaboration add‐on at readmission did not differ significantly across facility types (*p* = 0.296).

**TABLE 5:** Logistic regression analysis of factors associated with aspiration pneumonia.

Variable	Crude		Model 1		Model 2	
	OR (95% CI)	*p*	OR (95% CI)	*p*	OR (95% CI)	*p*
Facility type						
Super enhanced	0.858 (0.502–1.467)	0.576	0.865 (0.495–1.512)	0.611		
Enhanced	0.450 (0.224–0.903)	0.025	0.457 (0.225–0.926)	0.030		
Add‐on	0.804 (0.449–1.439)	0.463	0.817 (0.454–1.471)	0.501		
Basic	Ref.	Ref.	Ref.	Ref.		
Number of residents			1.096 (0.609–1.973)	0.759		
Mean care level			1.003 (0.996–1.010)	0.478		
History of AP					45.138 (16.937–120.292)	< 0.001
Aspiration					9.280 (4.215–20.116)	< 0.001
Occupational therapists						
0 persons					4.875 (1.708–13.909)	0.003
0 to < 1 person					1.176 (0.110–12.571)	0.893
1 to < 2 persons					1.823 (0.728–4.567)	0.200
≥ 2 persons					Ref.	Ref.
Certified care workers						
< 10					0.303 (0.035–2.623)	0.278
10 to < 20 persons					0.520 (0.080–3.360)	0.492
20 to < 30 persons					1.226 (0.201–7.474)	0.826
30 to < 40 persons					2.283 (0.321–16.214)	0.409
≥ 40 persons					Ref.	Ref.

*Note:* Model 1 was adjusted for the total number of residents and mean care level. Model 2 included the following variables: facility type; total number of residents; mean care level; history of AP; number of nursing staff; number of certified care workers; number of dietitians; number of dental hygienists; number of speech therapists; number of physical therapists; number of occupational therapists; presence of aspiration; presence of choking; number of residents receiving enteral nutrition; and number of staple food types. Variables were selected using backward stepwise logistic regression, and only significant predictors were retained in the final model.

Abbreviation: AP, aspiration pneumonia.

### 3.5 Association Between Facility Resources and AP

Compared with basic facilities, enhanced facilities showed a lower risk of AP (OR = 0.450, 95% CI = 0.224–0.903; *p* = 0.025). Super‐enhanced and add‐on categories were insignificant. Model 1 was adjusted for the number of residents and the mean care level. After adjustment, the significant association with enhanced facilities persisted (OR = 0.457, 95% CI = 0.225–0.926; *p* = 0.030). Model 2 was the multivariate final model in which a history of AP was strongly associated with current AP (OR = 45.138, 95% CI = 16.937–120.292; *p* < 0.001). In addition, aspiration at assessment was significantly associated with AP (OR = 9.280, 95% CI = 4.215–20.116; *p* < 0.001). Regarding staffing, facilities with 0 occupational therapists had a higher risk (OR = 4.875, 95% CI = 1.708–13.909; *p* = 0.003). For certified care workers, none of the categories differed from the reference of ≥ 40 workers. However, facility type was no longer retained in the final multivariable model after accounting for clinical and care‐process variables (Table 5).

To account for the reimbursement‐based add‐on structure of the Japanese LTCI system, no further adjustment was applied beyond Model 3 (Table S5). Within this fully adjusted model, facilities with a history of AP showed a markedly higher likelihood of having AP at the time of the survey (OR = 191.850, 95% CI 71.401–515.490; *p* < 0.001), as did those in which aspiration events occurred during the assessment period (OR = 19.827, 95% CI = 6.905–56.934; *p* < 0.001). Regarding staffing, facilities without occupational therapists were associated with a substantially higher odds of AP compared with those with ≥ 2 occupational therapists (OR = 10.694, 95% CI = 2.846–40.179; *p* < 0.001). In contrast, no significant associations were observed for certified care worker categories or other staffing groups after adjustment. Importantly, reimbursement‐based indicators of structured oral intake management were also associated with AP. Facilities claiming the oral intake maintenance add‐on I were associated with AP (OR = 1.086, 95% CI = 1.031–1.144; *p* = 0.002), whereas facilities claiming the oral intake maintenance add‐on II were also associated with AP (OR = 0.913, 95% CI = 0.866–0.963; *p* < 0.001). This contrasting pattern suggests a graded association according to the level of oral intake maintenance under the LTCI system.

A linear regression collinearity analysis was performed to examine the potential multicollinearity between the number of residents with aspirations and those with AP. Both variables showed very low variance inflation factors (VIF = 1.000), indicating no multicollinearity. The standardized coefficients were 0.376 for aspiration cases (*t* = 7.891, *p* < 0.001) and 0.866 for AP cases (*t* = 34.618, *p* < 0.001), suggesting that each variable contributed independently to the model.

## 4 Discussion

This study was conducted as a facility‐level analysis to examine the differences in AP prevention across long‐term care facilities. The analysis demonstrates how variations at the facility level may influence AP outcomes by focusing on organizational characteristics, support systems, and implemented interventions. These findings not only reveal disparities between facilities but also provide a theoretical basis for developing more effective strategies to prevent AP in institutional care settings.

AP is a major clinical and public health concern. More than 70% of hospitalizations for pneumonia are attributable to AP due to the country's rapidly aging population, underscoring its significance as a pressing issue requiring effective preventive and management strategies [14]. The Japanese Respiratory Society classifies pneumonia into community‐acquired pneumonia (CAP), hospital‐acquired pneumonia (HAP), and nursing and healthcare‐associated pneumonia (NHCAP) [15]. Although guidelines for these categories have been established, several aspects of AP overlap with those of HAP and NHCAP owing to patient characteristics, and the actual epidemiological situation remains insufficiently understood. Furthermore, recognizing AP is inherently challenging, leading to underdiagnosis and underreporting. Thus, available data are limited, incidence estimates lack accuracy, and associated risk factors cannot be adequately examined within restricted settings [3]. These gaps highlight the urgent need for further investigations focusing on AP, particularly at the facility level, to provide robust evidence for the development of effective preventive strategies.

AP arises from the interplay between impaired airway protection, reduced swallowing efficiency, and compromised host defense mechanisms. It is not a single event but rather a cumulative result of physiological aging, neurological dysfunction, and systemic frailty that disrupts the coordination between swallowing and respiration. Age‐related changes contribute substantially to AP susceptibility [16]. Age‐related sarcopenia causes the loss of oropharyngeal muscle mass and strength, resulting in delayed laryngeal elevation and incomplete glottic closure during swallowing [17]. Degenerative changes in the central nervous system further impair the coordination between the swallowing and respiratory centers [18], increasing the risk of aspiration during the inspiratory phase. In addition, diminished cough reflex sensitivity [19, 20] and reduced saliva production limit the clearance of aspirated material from the airway. Neurological and mechanical disorders also play a major role in this process. Stroke [21], Parkinson's disease [22], dementia [23], and other neurodegenerative conditions can disrupt voluntary and reflexive swallowing. Structural abnormalities, such as esophageal stricture, diverticulum, or postsurgical fibrosis, hinder bolus transit and predispose patients to residue accumulation and subsequent aspiration. Systemic and iatrogenic factors exacerbate this risk. Prolonged bed rest, malnutrition, and sedation reduce respiratory drive and muscular tone [24].

Our findings indicate that facilities with a history of AP are at higher risk of recurrent episodes. Consistent with previous reports, geriatric AP tends to recur and persist, reflecting ongoing microaspiration and long‐standing functional impairment; therefore, effective care must extend beyond antibiotics to include targeted interventions for swallowing dysfunction and airway defense support [25]. AP is characterized by pneumonia in individuals with impaired swallowing. As swallowing dysfunction is primarily attributed to age‐related physical decline [6], recovery from an acute episode of pneumonia does not alter the underlying functional impairment, causing a high likelihood of recurrence. Unlike acute diseases that typically result in sudden death, AP often follows a protracted course, in which repeated episodes progressively worsen the patient's condition and ultimately contribute to mortality [15]. Indeed, previous studies have demonstrated that individuals at risk of recurrent AP exhibit a markedly increased likelihood of death within 1 year, irrespective of the direct cause [26]. These observations support the prioritization of secondary prevention among residents with a history of AP.

Our facility‐level analysis revealed that institutions that reported a higher frequency of mis‐swallowing events, defined as overt choking or aspiration during meals, also exhibited a higher incidence of AP. This association is biologically and clinically plausible because mis‐swallowing and AP likely reflect related manifestations of underlying swallowing impairment and facility practices in facilities serving residents with persistent dysphagia and impaired airway protection [1, 2, 3]. At the facility level, shared determinants, including resident case‐mix such as frailty, dementia, post‐stroke status, staffing levels and skill mix, mealtime supervision practices, oral‐care routines, and the presence of formal swallowing‐support teams, can increase both mis‐swallowing and AP risk, which strengthens the observed correlation [2, 4, 5, 6]. Nevertheless, the overlap in definitions and measurements between mis‐swallowing, aspiration, and AP may introduce information bias. Facilities with stronger surveillance, such as routine mealtime observation, standardized bedside screening, and explicit AP‐coding protocols, are more likely to detect mis‐swallowing and classify pneumonia as aspiration‐related, which can inflate the association through differential misclassification and surveillance bias [3, 7]. These issues warrant cautious causal interpretation in ecological analyses and highlight the need to standardize operational definitions, staff training, and audit procedures across facilities.

From a practical standpoint, our findings support prioritizing facility‐wide AP‐related management pathways, including systematic screening with validated bedside tests and clear escalation criteria; mealtime observation with posture and texture modification and cueing; routine oral care bundles to reduce oropharyngeal bioburden; rapid response protocols after suspected AP that include temporary diet adjustment, hydration, and early assessment; and targeted training for high‐risk residents and caregivers [4, 5, 6, 8]. From a methodological standpoint, future facility‐level studies should adjust more comprehensively for resident case‐mix and institutional supports, should use incidence densities per resident‐day, evaluate mediation with mis‐swallowing as a mediator between facility practices and AP, implement sensitivity analyses that vary AP definitions, incorporate chart‐review substantiation, and stratify by surveillance intensity to estimate the contribution of measurement differences relative to true risk [1, 3, 7]. These steps can reduce bias, identify pathways from facility practices to AP, and inform scalable prevention strategies.

In the present study, occupational therapist availability was associated with AP occurrence at the facility level, whereas speech–language pathologist availability was not retained in the final model. This finding suggests that occupational therapists may play an important role in AP‐related management within long‐term care facilities. Although speech–language pathologists are typically responsible for swallowing assessment and rehabilitation in clinical practice, their availability did not show a corresponding association with AP occurrence in this facility‐level analysis. In the context of Japanese long‐term care settings, where speech–language pathologists are often not routinely available, occupational therapists may assume broader responsibilities related to feeding and swallowing safety, which may partly explain the observed association. Under these circumstances, occupational therapists often provide swallowing‐related interventions, including posture correction during meals, environmental and utensil modifications, and training to improve feeding independence and safety. Evidence suggests that multidisciplinary rehabilitation programs involving occupational therapist participation can enhance swallowing safety, reduce aspiration events, and lower the incidence of AP among frail older adults [27]. Facilities without occupational therapists may therefore lack consistent support for individualized AP‐related management, environmental adaptation, and caregiver training, which could contribute to the higher AP risk observed. These findings suggest that greater involvement of occupational therapists may represent a relevant consideration for AP‐related management and interprofessional coordination in institutional care environments where speech–language pathology resources are limited. In long‐term care facilities where speech–language pathology services are limited, establishing clear roles for occupational therapists in feeding safety, positioning, and environmental modifications can help address these gaps.

In addition to staffing, reimbursement‐based oral intake maintenance add‐ons under the LTCI system were also associated with AP in the present analysis. These add‐ons are administrative indicators that reflect whether a facility operates a structured framework for supporting oral intake in residents with eating or swallowing difficulties. Under the LTCI system, oral intake maintenance add‐on I is claimed when basic supportive measures for oral feeding are implemented, whereas the higher‐level add‐on II requires more comprehensive, multidisciplinary management, including formal care planning, monitoring, and coordinated involvement of rehabilitation and nutrition staff [28]. Facilities claiming only the lower‐level add‐on may include a higher proportion of residents at risk of AP but lack fully integrated management systems, whereas those claiming the higher‐level add‐on may reflect a more organized and intensive approach to oral intake support. Although these add‐ons were included primarily as control variables, the present findings suggest that how swallowing and feeding care is structured at the facility level may be relevant to AP occurrence and warrants further investigation.

An important and somewhat unexpected finding of the present study was that the number of nurses was not significantly associated with AP at the facility level. This result is counterintuitive given the central role of nurses in daily clinical care, airway management, and infection prevention. However, previous studies of long‐term care facilities have shown that staffing quality cannot be adequately captured by headcounts alone. Many nursing homes operate under suboptimal staffing conditions characterized by high turnover, extensive use of temporary agency staff, and a low proportion of professionally trained personnel, all of which have been associated with care quality and resident outcomes [29]. Under such conditions, the absolute number of nurses may not reflect the continuity, experience, or functional capacity of the nursing workforce [30], which may explain the lack of an observed association between nurse numbers and AP in this study. Consistent with this interpretation, a previous study reported that improvements in activities of daily living (ADL) and reductions in restraint use were associated with greater care time from unlicensed staff, whereas overall functional outcomes were largely unrelated to whether care was delivered by licensed or unlicensed personnel, except that greater ADL decline was observed among residents receiving more minutes of unlicensed care [31]. Moreover, previous work has shown that unit‐level staffing levels of both licensed and unlicensed staff are not necessarily associated with many care processes or outcomes, even though higher overall staffing is associated with more time devoted to direct resident care [31]. These observations support the notion that the intensity and organization of frontline caregiving may be more relevant to resident outcomes, including aspiration pneumonia risk, than staff headcounts alone.

## 5 Strengths and Limitations

To the best of our knowledge, this study is the first nationwide facility‐level analysis in Japan to examine how the organizational characteristics of long‐term care facilities relate to AP prevention. It leverages the 2024 JAGHSF census, providing broad geographic coverage and a large sampling frame of 3539 facilities, which enhances the external validity for institutional care settings.

This study has several limitations that merit consideration. First, the response rate was relatively low, and the analysis included only a subset of eligible facilities, which may limit representativeness. Second, the retrospective cross‐sectional design precludes causal inference and does not allow temporal ordering between facility practices and AP outcomes to be established. Third, the definitions of mis‐swallowing, aspiration events, and AP may vary across institutions, raising the possibility of misclassification. In particular, distinguishing AP from other types of pneumonia is often challenging, and the incidence of AP may therefore be underestimated. In addition, interactions among different staff categories were not examined in the present analysis. Although the OR was large, the wide CI indicated potential instability due to the small number of events, suggesting that this finding should be interpreted with caution. Moreover, staff turnover, which has been associated with resident outcomes in previous studies [32], was not available in the present dataset and therefore could not be evaluated. Given current workforce instability in Japan, future studies should examine whether staff turnover contributes to aspiration pneumonia risk. Future longitudinal studies are also needed to further clarify the determinants of AP risk.

## 6 Conclusions

This nationwide, facility‐level analysis of Japanese long‐term care facilities identified substantial between‐facility variations in AP outcomes that aligned with organizational characteristics, staffing resources, and AP‐related care practices. Facilities reporting more mis‐swallowing events had higher AP incidence, and a previous history of AP marked residents at high risk of recurrence, underscoring the need for targeted secondary prevention. Occupational therapists play a crucial role in cases where speech–language therapy services are limited. Although causal inference is limited by the cross‐sectional design, prospective multicenter evaluations using harmonized definitions and resident‐day denominators are warranted to evaluate the effectiveness and inform scalable implementation across long‐term care facilities.

## Funding

This work was supported by the Japan Science and Technology Agency (JST) through the Establishment of University Fellowships for the Creation of Science Technology Innovation (JPMJFS2102 to X.W.). Additional support was provided by the Japan Society for the Promotion of Science (JSPS) KAKENHI (22K19760, 24K02778, and 25K22899 to S.E.), Japan Agency for Medical Research and Development (25zf0127001h0005 to S.E.), Japan Science and Technology Agency (JST) Strategic International Collaborative Research Program (SICORP) (JPMJSC2308 to S.E.), the Research Funding for Longevity Sciences from the National Center for Geriatrics and Gerontology (25–29 to S.E.), and the Grant from the Japan Association of Geriatric Health Service Facilities (Dysphagia Statement Review Project to S.E.).

## Disclosure

The authors have nothing to report.

## Conflicts of Interest

The authors declare no conflicts of interest.

## Supporting information


**Figure S1:** Flowchart of the study. AP: aspiration pneumonia.

**Table S1:** Staff classification according to the presence of aspiration pneumonia (AP).
**Table S2:** Classification of staff according to the presence of aspirations.
**Table S3:** Staff classification according to the presence of choking.
**Table S4:** Distribution of nutrition‐ and oral‐management add‐ons across facility types.
**Table S5:** Logistic regression models for factors, including care‐related add‐ons, associated with aspiration pneumonia.

## Data Availability

The data that support the findings of this study are available on request from the corresponding author. The data are not publicly available due to privacy or ethical restrictions.
