# Supplementary figures and images for: Facility‐Level Factors Associated With Aspiration Pneumonia in Japanese Geriatric Health Service Settings: A Nationwide Cross‐Sectional Study
Source: Geriatr Gerontol Int. 2026 Feb 19;26(2):e70410. doi: 10.1111/ggi.70410 (PMC12917577; doi:10.1111/ggi.70410)

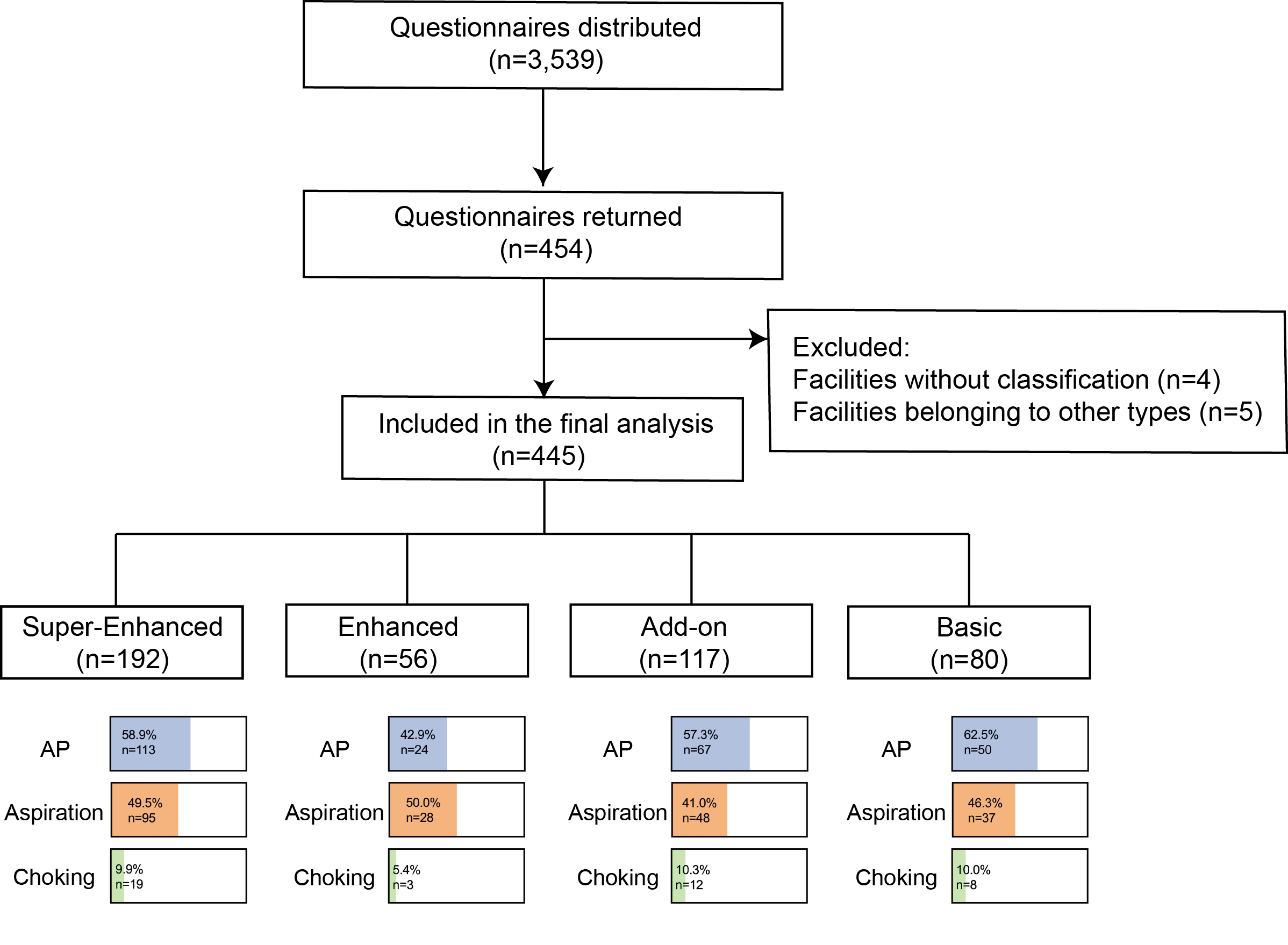

Supplement: Supplementary file 1 — Figure S1: Flowchart of the study. AP: aspiration pneumonia. [file GGI-26-0-s001.tif]
