# Supplementary material for: Facility‐Level Factors Associated With Aspiration Pneumonia in Japanese Geriatric Health Service Settings: A Nationwide Cross‐Sectional Study
Source: Geriatr Gerontol Int. 2026 Feb 19;26(2):e70410. doi: 10.1111/ggi.70410 (PMC12917577; doi:10.1111/ggi.70410)
Supplement: Supplementary file 2 — Table S1: Staff classification according to the presence of aspiration pneumonia (AP). Table S2: Classification of staff according to the presence of aspirations. Table S3: Staff classification according to the presence of choking. Table S4: Distribution of nutrition‐ and oral‐management add‐ons across facility types. Table S5: Logistic regression models for factors, including care‐related add‐ons, associated with aspiration pneumonia. [file GGI-26-0-s002.docx]

Table S1: Staff classification according to the presence of aspiration pneumonia (AP)

|  | AP | Non-AP | *P*-value |
| --- | --- | --- | --- |
| Number of residents (n) | 83.36±24.08 | 81.85±32.55 | 0.575 |
| Mean care level | 3.14±0.34 | 3.12±0.34 | 0.805 |
| Facility type (%, n) | | | 0.120 |
| Super Enhanced | 44.5(113) | 41.4(79) |  |
| Enhanced | 9.4(24) | 16.8(32) |  |
| Additional Support | 26.4(67) | 26.2(50) |  |
| Basic | 19.7(50) | 15.7(30) |  |
| Physicians (%, n) | | | 0.627 |
| <1 person | 9.2(23) | 10.1(19) |  |
| 1 to <2 persons | 82.1(206) | 83.6(158) |  |
| ≥2 persons | 8.8(22) | 6.3(12) |  |
| Dentists (%, n) | | | 0.256 |
| <1 person | 99.6(231) | 98.3(171) |  |
| 1 to <2 persons | 0.0(0) | 1.1(2) |  |
| ≥2 persons | 0.4(1) | 0.6(1) |  |
| Nursing staff (%, n) | | | 0.754 |
| <6 persons | 15.1(38) | 17.0(32) |  |
| 6 to <9 persons | 27.1(68) | 30.3(57) |  |
| 9 to <12 persons | 35.9(90) | 33.5(63) |  |
| ≥12 persons | 21.9(55) | 19.1(36) |  |
| Certified care workers (%, n) | | | 0.143 |
| <10 persons | 7.6(19) | 11.2(21) |  |
| 10 to <20 persons | 28.5(71) | 20.9(58) |  |
| 20 to <30 persons | 40.6(101) | 43.1(81) |  |
| 30 to <40 persons | 18.5(46) | 10.1(19) |  |
| ≥40 persons | 4.8(12) | 4.8(9) |  |
| Care staff (%, n) | | | 0.524 |
| <10 persons | 78.0(191) | 73.8(135) |  |
| 10 to <20 persons | 19.6(48) | 22.4(41) |  |
| ≥20 persons | 2.4(6) | 3.8(7) |  |
| Registered dietitians (%, n) | | | 0.507 |
| 0 persons | 1.6(4) | 2.6(5) |  |
| 0 to <1 person | 0.4(1) | 1.6(3) |  |
| 1 to <2 persons | 52.2(132) | 51.1(97) |  |
| ≥2 persons | 45.8(116) | 44.7(85) |  |
| Nutritionists (%, n) | | | 0.482 |
| 0 persons | 84.8(196) | 80.9(140) |  |
| 0 to <1 person | 11.3(26) | 13.3(23) |  |
| 1 to <2 persons | 0.4(1) | 0.0(0) |  |
| ≥2 persons | 3.5(8) | 5.8(10) |  |
| Dental hygienists (%, n) | | | 0.595 |
| 0 persons | 88.1(208) | 85.1(149) |  |
| 0 to <1 person | 1.3(3) | 0.6(1) |  |
| 1 to <2 persons | 8.9(21) | 11.4(20) |  |
| ≥2 persons | 1.7(4) | 2.9(5) |  |
| Speech-language pathologists (%, n) | | | 0.424 |
| 0 persons | 52.1(125) | 49.4(88) |  |
| 0 to <1 person | 2.9(7) | 3.9(7) |  |
| 1 to <2 persons | 36.7(88) | 33.7(60) |  |
| ≥2 persons | 8.3(20) | 12.9(23) |  |
| Physical therapists (%, n) | | | 0.941 |
| 0 persons | 4.4(11) | 5.4(10) |  |
| 0 to <1 person | 0.8(2) | 1.1(2) |  |
| 1 to <2 persons | 15.2(38) | 15.8(29) |  |
| ≥2 persons | 79.6(199) | 77.7(143) |  |
| Occupational therapists (%, n) | | | 0.702 |
| 0 persons | 14.2(35) | 12.0(22) |  |
| 0 to <1 person | 2.8(7) | 1.6(3) |  |
| 1 to <2 persons | 24.3(60) | 23.4(43) |  |
| ≥2 persons | 58.7(145) | 63.0(116) |  |

Table S2: Classification of staff according to the presence of aspirations.

|  | Aspiration | Non-aspiration | *P*-value |
| --- | --- | --- | --- |
| Number of residents (n) | 96.09±24.42 | 92.38±27.69 | 0.128 |
| Mean care level | 3.13±0.33 | 3.14±0.34 | 0.107 |
| Facility type (%, n) | | | 0.501 |
| Super Enhanced | 45.7（95） | 40.9（97） |  |
| Enhanced | 13.5（28） | 11.8（28） |  |
| Additional Support | 23.1（48） | 29.1（69） |  |
| Basic | 17.8（37） | 18.1（43） |  |
| Physicians (%, n) | | | 0.555 |
| <1 person | 9.2（19） | 9.9（23） |  |
| 1 to <2 persons | 81.6（169） | 83.7（195） |  |
| ≥2 persons | 9.2（19） | 6.4（15） |  |
| Dentists (%, n) | | | 0.412 |
| <1 person | 99.5（189） | 98.6（213） |  |
| 1 to <2 persons | 0.0（0） | 0.9（2） |  |
| ≥2 persons | 0.5（1） | 0.5（1） |  |
| Nursing staff (%, n) | | | 0.648 |
| <6 persons | 15.0（31） | 16.8（39） |  |
| 6 to <9 persons | 27.1（56） | 29.7（69） |  |
| 9 to <12 persons | 34.8（72） | 34.9（81） |  |
| ≥12 persons | 23.2（48） | 18.5（43） |  |
| Certified care workers (%, n) | | | 0.250 |
| <10 persons | 8.7（18） | 9.5（22） |  |
| 10 to <20 persons | 28.6（59） | 30.3（70） |  |
| 20 to <30 persons | 38.3（79） | 44.6（103） |  |
| 30 to <40 persons | 18.4（38） | 11.7（27） |  |
| ≥40 persons | 5.8（12） | 3.9（9） |  |
| Care staff (%, n) | | | 0.871 |
| <10 persons | 76.2（154） | 76.1（172） |  |
| 10 to <20 persons | 20.3（41） | 21.2（48） |  |
| ≥20 persons | 3.5（7） | 2.7（6） |  |
| Registered dietitians (%, n) | | | 0.573 |
| 0 persons | 2.4（5） | 1.7（4） |  |
| 0 to <1 person | 0.5（1） | 1.3（3） |  |
| 1 to <2 persons | 49.3（102） | 53.8（127） |  |
| ≥2 persons | 47.8（99） | 43.2（102） |  |
| Nutritionists (%, n) | | | 0.813 |
| 0 persons | 83.2（158） | 83.2（178） |  |
| 0 to <1 person | 12.1（23） | 12.1（26） |  |
| 1 to <2 persons | 0.0（0） | 0.5（1） |  |
| ≥2 persons | 4.7（9） | 4.2（9） |  |
| Dental hygienists (%, n) | | | 0.227 |
| 0 persons | 85.5（165） | 88.1（192） |  |
| 0 to <1 person | 0.5（1） | 1.4（3） |  |
| 1 to <2 persons | 10.4（20） | 9.6（21） |  |
| ≥2 persons | 3.6（7） | 0.9（2） |  |
| Speech-language pathologists (%, n) | | | 0.050 |
| 0 persons | 46.4（91） | 55.0（122） |  |
| 0 to <1 person | 2.0（4） | 4.5（10） |  |
| 1 to <2 persons | 41.8（82） | 29.7（66） |  |
| ≥2 persons | 9.7（19） | 10.8（24） |  |
| Physical therapists (%, n) | | | 0.447 |
| 0 persons | 3.4（7） | 6.1（14） |  |
| 0 to <1 person | 0.5（1） | 1.3（3） |  |
| 1 to <2 persons | 16.0（33） | 14.9（34） |  |
| ≥2 persons | 80.1（165） | 77.6（177） |  |
| Occupational therapists (%, n) | | | 0.422 |
| 0 persons | 10.8（22） | 15.4（35） |  |
| 0 to <1 person | 3.0（6） | 1.8（4） |  |
| 1 to <2 persons | 23.2（47） | 24.6（56） |  |
| ≥2 persons | 63.1（128） | 58.3（133） |  |

Table S3: Staff classification according to the presence of choking.

|  | Choking | Non-choking | *P-*value |
| --- | --- | --- | --- |
| Number of residents (n) | 94.44±13.15 | 94.02±27.25 | 0.923 |
| Mean care level | 3.10±0.28 | 3.13±0.34 | 0.548 |
| Facility type (%, n) | | | 0.762 |
| Super Enhanced | 45.2(19) | 42.9(173) |  |
| Enhanced | 7.1(3) | 13.2(53) |  |
| Additional Support | 28.6(12) | 26.1(105) |  |
| Basic | 19.0(8) | 17.9(72) |  |
| Physicians (%, n) | | | 0.182 |
| <1 person | 4.8(2) | 10.1(40) |  |
| 1 to <2 persons | 92.9(39) | 81.7(325) |  |
| ≥2 persons | 2.4(1) | 8.3(33) |  |
| Dentists (%, n) | | | 0.807 |
| <1 person | 100.0(39) | 98.9(363) |  |
| 1 to <2 persons | 0.0(0) | 0.5(2) |  |
| ≥2 persons | 0.0(0) | 0.5(2) |  |
| Nursing staff (%, n) | | | 0.377 |
| <6 persons | 7.3(3) | 16.8(67) |  |
| 6 to <9 persons | 31.7(13) | 28.1(112) |  |
| 9 to <12 persons | 34.1(14) | 34.9(139) |  |
| ≥12 persons | 26.8(11) | 20.1(80) |  |
| Certified care workers (%, n) | | | 0.023 |
| <10 persons | 0.0(0) | 10.1(40) |  |
| 10 to <20 persons | 22.0(9) | 30.3(120) |  |
| 20 to <30 persons | 61.0(25) | 39.6(157) |  |
| 30 to <40 persons | 17.1(7) | 14.6(58) |  |
| ≥40 persons | 0.0(0) | 5.3(21) |  |
| Care staff (%, n) | | | 0.258 |
| <10 persons | 85.4(35) | 75.2(291) |  |
| 10 to <20 persons | 14.6(6) | 21.4(83) |  |
| ≥20 persons | 0.0(0) | 3.4(13) |  |
| Registered dietitians (%, n) | | | 0.929 |
| 0 persons | 2.4(1) | 2.0(8) |  |
| 0 to <1 person | 0.0(0) | 1.0(4) |  |
| 1 to <2 persons | 52.4(22) | 51.6(207) |  |
| ≥2 persons | 45.2(19) | 45.4(182) |  |
| Nutritionists (%, n) | | | 0.897 |
| 0 persons | 79.5(31) | 83.6(305) |  |
| 0 to <1 person | 15.4(6) | 11.8(43) |  |
| 1 to <2 persons | 0.0(0) | 0.3(1) |  |
| ≥2 persons | 5.1(2) | 4.4(16) |  |
| Dental hygienists (%, n) | | | 0.870 |
| 0 persons | 89.7(35) | 86.6(322) |  |
| 0 to <1 person | 0.0(0) | 1.1(4) |  |
| 1 to <2 persons | 7.7(3) | 10.2(38) |  |
| ≥2 persons | 2.6(1) | 2.2(8) |  |
| Speech-language pathologists (%, n) | | | 0.580 |
| 0 persons | 57.5(23) | 50.3(190) |  |
| 0 to <1 person | 0.0(0) | 3.7(14) |  |
| 1 to <2 persons | 32.5(13) | 35.7(135) |  |
| ≥2 persons | 10.0(4) | 10.3(39) |  |
| Physical therapists (%, n) | | | 0.605 |
| 0 persons | 4.9（2） | 4.8（19） |  |
| 0 to <1 person | 0.0（0） | 1.0（4） |  |
| 1 to <2 persons | 22.0（9） | 14.8（58） |  |
| ≥2 persons | 73.2（30） | 79.4（312） |  |
| Occupational therapists (%, n) | | | 0.400 |
| 0 persons | 5.0（2） | 14.1（55） |  |
| 0 to <1 person | 2.5（1） | 2.3（9） |  |
| 1 to <2 persons | 22.5（9） | 24.0（94） |  |
| ≥2 persons | 70.0（28） | 59.6（233） |  |

Table S4: Distribution of nutrition- and oral-management add-ons across facility types.

|  | Super-enhanced | Enhanced | Add-on | Basic | *P* value |
| --- | --- | --- | --- | --- | --- |
| Nutritional management enhancement | 70.00(0.00-86.00) | 71.70(0.00-89.75) | 30.50(0.00-78.00) * | 0.00(0.00-68.00) ** # | <0.001 |
| Oral intake transition | 0.00(0.00-0.00) | 0.00(0.00-0.00) | 0.00(0.00-0.00) | 0.00(0.00-0.00) | 0.153 |
| Oral intake maintenance I | 10.00(1.00-23.00) | 2.00(0.00-19.00) | 3.00(0.00-15.75) * | 0.00(0.00-5.00) ** # | <0.001 |
| Oral intake maintenance II | 8.00(0.00-22.00) | 0.00(0.00-14.00) | 0.00(0.00-6.75) ** | 0.00(0.00-0.00) ** # | <0.001 |
| Oral hygiene management I | 0.00(0.00-0.00) | 0.00(0.00-0.00) | 0.00(0.00-0.00) | 0.00(0.00-0.00) | 0.369 |
| Oral hygiene management II | 6.50(0.0-66.25) | 0.00(0.00-37.00) * | 0.00(0.00-17.25) ** | 0.00(0.00-0.00) ** | <0.001 |
| Nutritional collaboration add-on readmission | 0.00(0.00-0.00) | 0.00(0.00-0.00) | 0.00(0.00-0.00) | 0.00(0.00-0.00) | 0.296 |

Data are presented as mean ± SD. * *p* < 0.05, ** *p* < 0.01 compared with super enhanced type; # *p* < 0.05, compared with enhanced type. AP: aspiration pneumonia.

Table S5: Logistic regression models for factors, including care-related add-ons, associated with aspiration pneumonia.

| Variable | Crude | | Model 1 | | Model 2 | | Model 3 | |
| --- | --- | --- | --- | --- | --- | --- | --- | --- |
|  | OR (95%CI) | *P*-value | OR (95%CI) | *P*-value | OR (95%CI) | *P*-value | OR (95%CI) | *P*-value |
| **Facility type** |  |  |  |  |  |  |  |  |
| Super Enhanced | 0.858(0.502-1.467) | 0.576 | 0.865(0.495-1.512) | 0.611 |  |  |  |  |
| Enhanced | 0.450(0.224-0.903) | 0.025 | 0.457(0.225-0.926) | 0.030 |  |  |  |  |
| Add-on | 0.804(0.449-1.439) | 0.463 | 0.817(0.454-1.471) | 0.501 |  |  |  |  |
| Basic | Ref. | Ref. | Ref. | Ref. |  |  |  |  |
| **Number of residents** |  |  | 1.096(0.609-1.973) | 0.759 |  |  |  |  |
| **Mean care level** |  |  | 1.003(0.996-1.010) | 0.478 |  |  |  |  |
| **History of AP** |  |  |  |  | 45.138(16.937-120.292) | <0.001 | 191.850 (71.401-515.490) | <0.001 |
| **Aspiration** |  |  |  |  | 9.280(4.215-20.116) | <0.001 | 19.827(6.905-56.934) | <0.001 |
| **Occupational therapists** |  |  |  |  |  |  |  |  |
| 0 persons |  |  |  |  | 4.875(1.708-13.909) | 0.003 | 10.694(2.846-40.179) | <0.001 |
| 0 to <1 person |  |  |  |  | 1.176(0.110-12.571) | 0.893 | 0.683(0.029-15.880) | 0.812 |
| 1 to <2 persons |  |  |  |  | 1.823(0.728-4.567) | 0.200 | 1.353(0.452-4.049) | 0.588 |
| ≥2 persons |  |  |  |  | Ref. | Ref. | Ref. | Ref. |
| **Certified care workers** |  |  |  |  |  |  |  |  |
| <10 persons |  |  |  |  | 0.303(0.035-2.623) | 0.278 | 0.103(0.005-1.990) | 0.133 |
| 10 to <20 persons |  |  |  |  | 0.520(0.080-3.360) | 0.492 | 0.627(0.053-7.419) | 0.711 |
| 20 to <30 persons |  |  |  |  | 1.226(0.201-7.474) | 0.826 | 1.275(0.126-12.937) | 0.837 |
| 30 to <40 persons |  |  |  |  | 2.283(0.321-16.214) | 0.409 | 2.004(0.163-24.614) | 0.587 |
| ≥40 persons |  |  |  |  | Ref. | Ref. | Ref. | Ref. |
| Oral intake maintenance add-on (I) |  |  |  |  |  |  | 1.086(1.031-1.144) | 0.002 |
| Oral intake maintenance add-on (II) |  |  |  |  |  |  | 0.913(0.866-0.963) | <0.001 |

Model 1 was adjusted for the total number of residents and mean care level.

Model 2 included the following variables: facility type; total number of residents; mean care level; history of AP; number of nursing staff; number of certified care workers; number of dietitians; number of dental hygienists; number of speech therapists; number of physical therapists; number of occupational therapists; presence of aspiration; presence of choking; number of residents receiving enteral nutrition; and number of staple food types.

Model 3 further extended Model 2 by including reimbursement-based nutritional and oral management add-ons: nutritional management enhancement, oral intake maintenance I and II, and oral hygiene management.

Variables were selected using backward stepwise logistic regression, and only significant predictors were retained in the final model. AP: aspiration pneumonia.
